# Analysis and Self-Calibration Method for Asynchrony between Sensors in Rotation INS

**DOI:** 10.3390/s18092921

**Published:** 2018-09-03

**Authors:** Jie Sui, Lei Wang, Tao Huang, Qi Zhou

**Affiliations:** 1 School of Instrumentation Science and Opto-electronics Engineering, Beihang University, Beijing 100191, China; suijie1224@buaa.edu.cn (J.S.); huang_tao@buaa.edu.cn (T.H.); 2 National Key Laboratory of Science and Technology on Aircraft Control, Xi’an 710065, China; kjb@facri.com

**Keywords:** accelerometer, angular encoder, asynchrony, fiber optic gyroscope (FOG), rotation inertial navigation system (RINS), self-calibration

## Abstract

The gyroscope, accelerometer and angular encoder are the most important components in a dual-axis rotation inertial navigation system (RINS). However, there are asynchronies among the sensors, which will thus lead to navigation errors. The impact of asynchrony between the gyroscope and angular encoder on the azimuth error and the impact of asynchrony between the gyroscope and accelerometer on the velocity error are analyzed in this paper. A self-calibration method based on navigation errors is proposed based on the analysis above. Experiments show that azimuth and velocity accuracy can be improved by compensating the asynchronies.

## 1. Introduction

The inertial navigation system (INS) employs three gyroscopes and three accelerometers to measure angular velocities and specific force, respectively, and obtains the real-time velocities, positions and attitudes of the vehicle according to the dead reckoning principle. Gyroscope bias and accelerometer bias are the major error sources of INS. To reduce the impacts of those important error sources and improve navigation accuracy, the rotation modulation technique has been researched extensively and profoundly in recent years [1,2,3].

The RINS employs the rotation modulation technique to reduce the impact of gyroscope and accelerometer bias and improve navigation accuracy. Nowadays, RINSs are widely utilized on ships and submarines, and RINSs for aircraft and missiles are being studied [4,5,6,7,8]. The widely used and researched RINS can be divided into several categories according to different classifications. In terms of the rotation mode, the RINS can be divided into continuous rotation RINS and discontinuous rotation RINS [9,10,11,12,13]. The constant errors of the sensors are modulated into sine or cosine form by the continuous rotation scheme [9,10]. An 8-position discontinuous rotation scheme is proposed in [12,13]. With respect to the rotation axis, the RINS can be divided into single-axis RINS, dual-axis RINS and triple-axis RINS [9,10,11,12,13,14,15]. The differences between single-axis RINS and dual-axis RINS are studied in [15]. The former cannot modulate the biases of the inertial sensor with the input axis coinciding with the rotation axis and the later can modulate the constant errors of all sensors by the appropriate rotation scheme [12,13,14].

The rotation modulation technique can reduce the impacts of gyro scope bias and accelerometer bias. However, the influences of other error sources may be amplified at the same time. The error sources include the calibration factor error of the gyroscope, misalignment an gles of the gyroscope, misalignment angles of the accelerometer, inner level-arm error, non-orthogonal angles of gimbals, etc. There are some papers about these error sources, and appropriate calibration and compensation methods are proposed to reduce the impact of these errors and improve navigation accuracy [1,15,16,17,18,19].

The inertial navigation algorithm is based on ideal measurements from sensors, while in the actual INS, time-asynchrony between different sensors is inevitable. The causes of the asynchronies are the phase-shift characteristic inconsistency between different sensors, and signal transmission delays [20,21]. The impact of time-asynchrony between laser gyroscopes on strap-down inertial navigation system (SINS) attitude computation accuracy is analyzed in [22] and a compensation method is proposed in [23]. Both articles only focus on the asynchrony between gyroscopes. A calibration algorithm for time-delay of accelerometers is proposed in [24]. A compensation method for time-delay of accelerometers in SINS is proposed in [20]. Yan provides a method to identify the time-asynchrony parameters between gyroscopes and accelerometers [21]. The methods in the references need complex calibration procedures and a swaying turntable. In this paper, a self-calibration method for asynchronies between different sensors is proposed.

The rest of the paper is arranged as follows: Section 2 introduces the structure of the dual-axis RINS, the definition of frames, the attitude calculation method, and the rotation scheme. The analysis of the asynchrony between the gyroscope and angular encoder is given in Section 3. The analysis of the asynchrony between the gyroscope and accelerometer is conducted in Section 4. Section 5 provides the conclusions of the study.

## 2. Introduction to the RINS

The RINS analyzed in this paper has some features in term of structure, frames, attitude calculation method, and rotation schemes. These characteristics are introduced below.

### 2.1. Structure of the RINS

**Figure 1:**
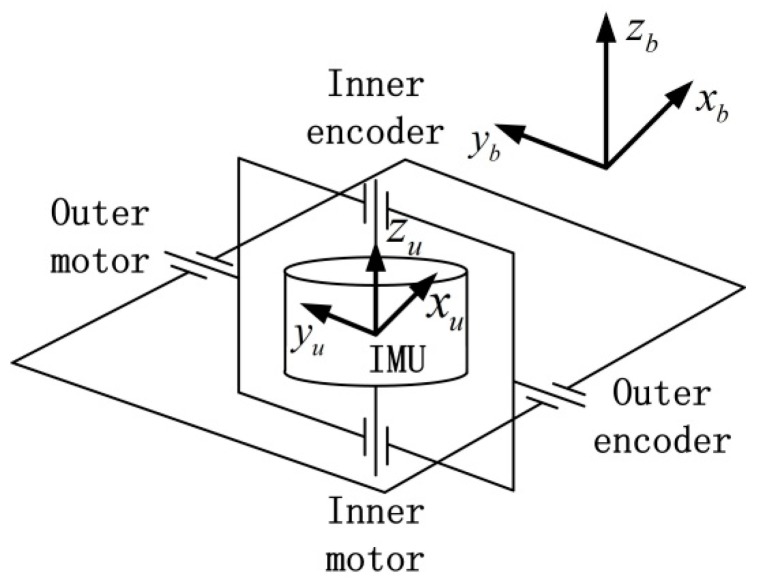
Structure of the dual-axis RINS.

The structure of the experimental dual-axis RINS is shown in Figure 1. The inertial measurement unit (IMU) of the RINS consists of orthogonally mounted tri-axis fiber optic gyroscopes (FOGs) and three quartz flexure accelerometers. The RINS contains an inner frame and an outer frame. Either frame contains a motor and an absolute angular encoder, which are mounted coaxially. The IMU can rotate along the inner frame axis continuously, and the inner frame can rotate along the outer frame. The angles rotated are given by the angular encoders. Table 1 shows the specifications of the main components of the experimental RINS. The parameters are given by the datasheets of the sensors and are confirmed by tests beforehand.

### 2.2. Definition of Frames

There are three frames used in the experimental RINS: the IMU frame, the body frame and the navigation frame. Their definitions are given below:

IMU frame ($x_{u}$, $y_{u}$, $z_{u}$): a right–handed orthogonal coordinate system, where, $z_{u}$ is aligned with the direction of the inner motor axis, $x_{u}$ points to the projection direction of the x-axis of the gyroscope in the $x_{u}-o-y_{u}$ flat, and $y_{u}$ is perpendicular to $x_{u}$ and $z_{u}$.

Body frame ($x_{b}$, $y_{b}$, $z_{b}$): an orthogonal coordinate system, where, $x_{b}$, $y_{b}$, and $z_{b}$ are aligned with the transverse axis, longitudinal axis, and perpendicular axis of the body respectively.

Navigation frame ($x_{n}$, $y_{n}$, $z_{n}$): a local level coordinate system, where, $z_{n}$ is aligned with the geodetic vertical, $y_{n}$ is aligned with the north direction, and $x_{n}$ is aligned with the east direction.

As shown in Figure 1, it should be noticed that the outer motor is coaxial with $y_{b}$ axis of the body frame. The IMU frame rotates with the motors and it is consistent with the body frame when the outputs of the angular encoders are zero.

### 2.3. Attitude Calculation Method

**Figure 2:**
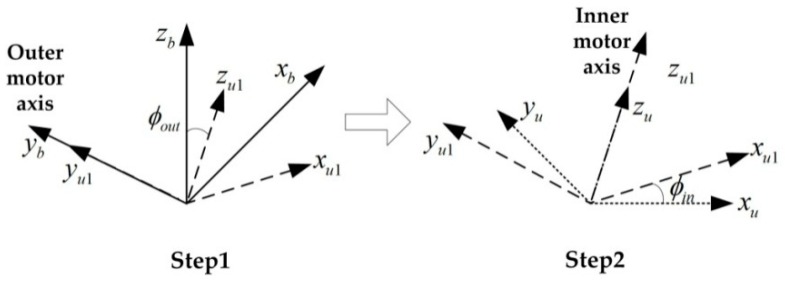
Relationship between body frame and IMU frame.

The velocity and position of the vehicle and the rotation matrix from IMU frame to navigation frame, i.e., $C_{u}^{n}$ can be obtained by the strap-down navigation algorithm based on the IMU measurements. The angular encoders provide the rotation matrix from body frame to IMU frame, i.e., $C_{b}^{u}$. The relationship between body frame and IMU frame is shown in Figure 2. The body frame can be transferred to IMU frame by two rotation steps. First step, rotating along outer motor axis for $\phi_{out}$; second step, rotating along inner motor axis for $\phi_{in}$. As the outer motor axis is aligned with the $y_{b}$ axis of body frame and the inner motor axis is aligned with the $z_{u}$ axis of IMU frame, the rotation matrix can be obtained by Equation (1):

(1) $$C_{b}^{u}=\left[\begin{array}{ccc} \cos(\phi_{in}) & \sin(\phi_{in}) & 0\\ -\sin(\phi_{in}) & \cos(\phi_{in}) & 0\\ 0 & 0 & 1 \end{array}\right]\left[\begin{array}{ccc} \cos(\phi_{out}) & 0 & -\sin(\phi_{out})\\ 0 & 1 & 0\\ \sin(\phi_{out}) & 0 & \cos(\phi_{out}) \end{array}\right]$$

where $\phi_{in}$ represents the output of the inner angular encoder, and $\phi_{out}$ represents the output of the outer angular encoder.

Then the attitude of the body frame relative to the navigation frame ($C_{b}^{n}$) is obtained according to Equation (2):

(2) $$C_{b}^{n}=C_{u}^{n}\cdot C_{b}^{u}$$

### 2.4. Rotation Scheme

**Figure 3:**
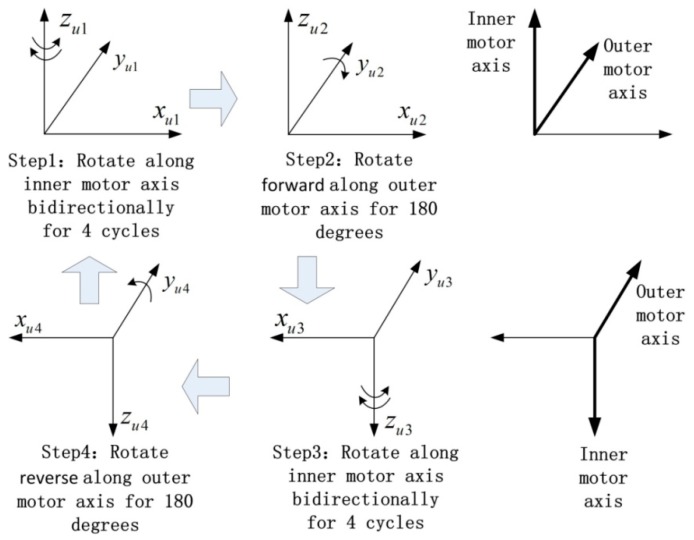
Process of rotation scheme.

The rotation scheme adopted in the experimental dual-axis RINS in this paper is shown in Figure 3. The scheme combines the continuous rotation scheme and the discontinuous rotation scheme to modulate the gyroscope bias and the accelerometer bias [16]. By the rotation technique, the drifts in the body frame are modulated into sine or cosine forms with an average value of zero in theory. Therefore, the navigation accuracy can be improved.

Firstly, the IMU rotates along the inner motor axis bidirectionally for four cycles with the angular velocity of 6°/s. Secondly, the IMU rotates forward along the outer motor axis for 180° with the angular velocity of 30°/s. Thirdly, the IMU rotates along the inner motor axis bidirectionally for four cycles with the angular velocity of 6°/s. Fourthly, the IMU rotates reversely along the outer motor axis for 180° with the angular velocity of 30°/s, and then goes back to the start status of the first step. The whole modulation scheme lasts 972 s.

## 3. Analysis of Asynchrony between Gyroscope and Angular Encoder

**Figure 4:**
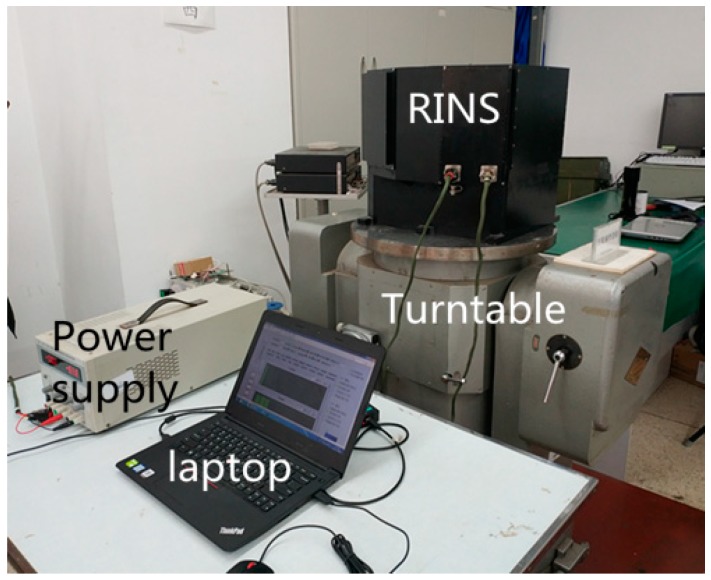
RINS and associated experimental equipment.

**Figure 5:**
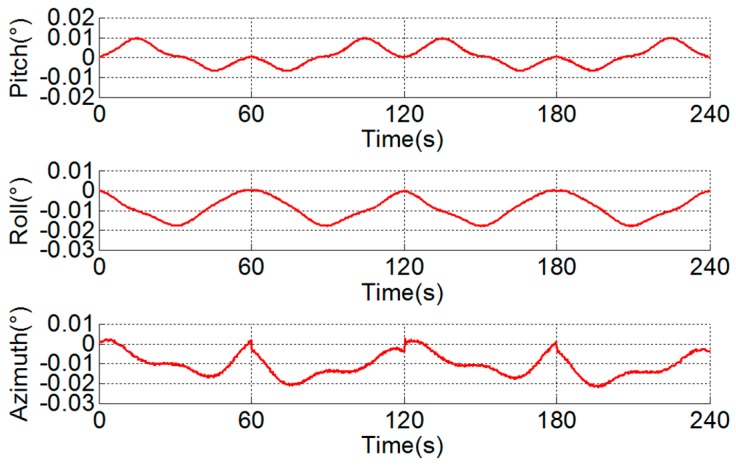
Original attitude outputs of RINS.

An experiment is conducted to research the asynchrony between the gyroscope and angular encoder. As shown in Figure 4, the dual-axis RINS is placed on a turntable with the attitude of East-North-Up. The IMU rotates according to the scheme provided in Section 2. In the first step of the scheme, the IMU rotates along the inner motor axis bidirectionally. The attitude outputs of the body frame in the first 240 s are shown in Figure 5.

**Figure 6:**
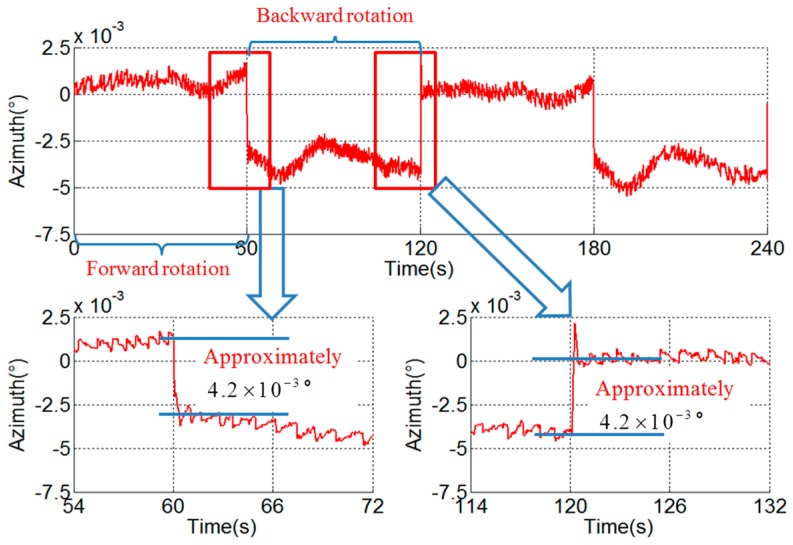
Stair error of azimuth (ω = 6°/s).

The pitch and roll outputs are mixed with the error related to the rotation period caused by vortex motion of the axis, and the error is calibrated and compensated by the method proposed in [25]. The rotation period error in the azimuth is caused by the angular encoder installation error, i.e., error caused by the eccentricity, swash, and distortion of the ring and can be compensated by the method given in [26]. However, there remains a stair error of the azimuth during the turn-round of the IMU, as shown in Figure 6. It can be seen that the stair error is about $4.2\times 10^{-3}$° and is not analyzed in existing literature.

### 3.1. Simplification of Attitude Calculation Method

According to the attitude tracking algorithm, the azimuth of the body frame can be obtained from the rotation matrix from body frame to navigation frame, i.e., $C_{b}^{n}$. In order to analyze the cause of the azimuth stair more clearly, the attitude calculation method will be simplified based on the experimental conditions.

The dual-axis RINS is placed with the attitude of East-North-Up, and the inner angular encoder output is zero during the first step of the modulation scheme. Therefore, the pitch and roll angles of the IMU are almost zero. The rotation matrix from IMU frame to navigation frame, i.e., $C_{u}^{n}$, can be simplified according to Equation (3). The rotation matrix from body frame to IMU frame, i.e., $C_{b}^{u}$, can be simplified according to Equation (4):

(3) $$\begin{array}{cc} C_{u}^{n} & =\left[\begin{array}{ccc} \cos\psi & -\sin\psi & 0\\ \sin\psi & \cos\psi & 0\\ 0 & 0 & 1 \end{array}\right]\cdot \left[\begin{array}{ccc} 1 & 0 & 0\\ 0 & \cos\theta & -\sin\theta \\ 0 & \sin\theta & \cos\theta \end{array}\right]\cdot \left[\begin{array}{ccc} \cos\gamma & 0 & \sin\gamma \\ 0 & 1 & 0\\ -\sin\gamma & 0 & \cos\gamma \end{array}\right]\\ & =R_{z}(-\psi )\cdot R_{x}(-\theta )\cdot R_{y}(-\gamma )\\ & =R_{z}(-\psi ) \end{array}$$

where θ, γ, ψ are the pitch, roll and azimuth angles of IMU frame:

(4) $$\begin{array}{cc} C_{b}^{u} & =\left[\begin{array}{ccc} \cos(\phi_{in}) & \sin(\phi_{in}) & 0\\ -\sin(\phi_{in}) & \cos(\phi_{in}) & 0\\ 0 & 0 & 1 \end{array}\right]\left[\begin{array}{ccc} \cos(\phi_{out}) & 0 & -\sin(\phi_{out})\\ 0 & 1 & 0\\ \sin(\phi_{out}) & 0 & \cos(\phi_{out}) \end{array}\right]\\ & =R_{z}(\phi_{in})\cdot R_{y}(\phi_{out})\\ & =R_{z}(\phi_{in}) \end{array}$$

Then the rotation matrix from body frame to navigation frame can be simplified according Equation (5).The azimuth of the body frame is $\psi -\phi_{in}$:

(5) $$\begin{array}{cc} C_{b}^{n} & =C_{u}^{n}\cdot C_{b}^{u}\\ & =R_{z}(-\psi )\cdot R_{z}(\phi_{in})\\ & =R_{z}(-\psi +\phi_{in}) \end{array}$$

**Figure 7:**
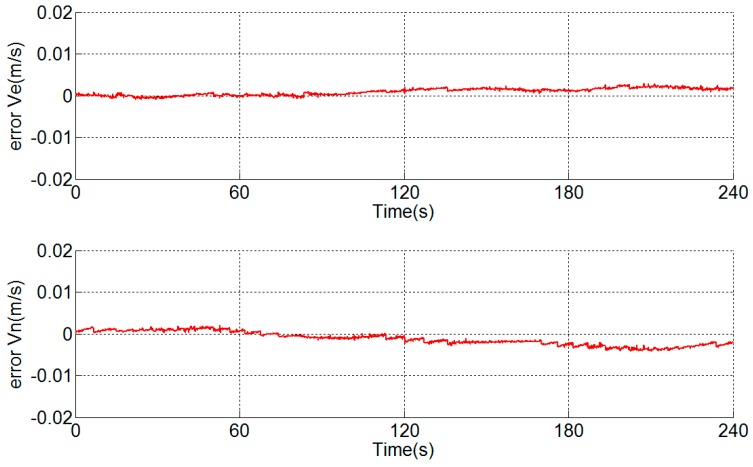
Velocity errors.

There is no additional velocity error during and after the turn-round of the IMU (see Figure 7). As the IMU frame attitude stair error during the turn-round will lead to velocity error during and after the turn-round [27], it is concluded that the IMU frame attitude accuracy is normal during the turn-round. The causes of the stair error of azimuth exist in the angular encoder or between the gyroscope and the angular encoder, i.e., the hysteresis error of the angular encoder or the asynchrony between those two sensors. They will be analyzed respectively below.

### 3.2. Hysteresis Error

The hysteresis error is one of the important errors of an angular encoder. The relationship among the observed value, theoretical value and hysteresis error of the angular encoder is shown in Equation (6):

(6) $$\phi_{in}^{o}=\{\begin{array}{l} \phi_{in}^{t}-\Delta \phi_{fh}\;(\mathrm{Forward})\\ \phi_{in}^{t}-\Delta \phi_{bh}\;(\mathrm{Backward}) \end{array}$$

where $\phi_{in}^{o}$ is the observed value of the angular encoder, $\phi_{in}^{t}$ is the theoretical value of the angular encoder, $\Delta \phi_{fh}$ is the hysteresis error of the angular encoder during the forward rotation, $\Delta \phi_{bh}$ is the hysteresis error of the angular encoder during the backward rotation.

Equation (5) should be modified to Equation (7) because of the hysteresis error. It can be seen from Equation (7) that the hysteresis error of the angular encoder will lead to the stair error of azimuth during the turn-round of the IMU, and the stair error is $\Delta \phi_{fh}-\Delta \phi_{bh}$.

(7) $$C_{b}^{n}=\{\begin{array}{l} R_{z}(-\psi +\phi_{in}^{t}-\Delta \phi_{fh})\;(\mathrm{Forward})\\ R_{z}(-\psi +\phi_{in}^{t}-\Delta \phi_{bh})\;(\mathrm{Backward}) \end{array}$$

### 3.3. Asynchrony between Gyroscope and Angular Encoder

The asynchrony between those two sensors will cause the stair error too. It is supposed that the inner angular encoder data is Δt earlier than gyroscope data. The relationship between observed value and theoretical value of the angular encoder is shown in Equation (8):

(8) $$\phi_{in}^{o}=\{\begin{array}{l} \phi_{in}^{t}+\omega \Delta t\;(\mathrm{Forward})\\ \phi_{in}^{t}-\omega \Delta t\;(\mathrm{Backward}) \end{array}$$

where ω is the angular velocity of the IMU in the first step of the rotation scheme, namely 6°/s, Δt is the asynchrony between the angular encoder and gyroscope, $\phi_{in}^{t}$ is the theoretical angular encoder value at the same time with the gyroscope data, $\phi_{in}^{o}$ is the angular encoder value at the response time of the encoder.

Then Equation (5) should be modified to Equation (9) because of the asynchrony between two sensors:

(9) $$C_{b}^{n}=\{\begin{array}{l} R_{z}(-\psi +\phi_{in}^{t}+\omega \Delta t)\;(\mathrm{Forward})\\ R_{z}(-\psi +\phi_{in}^{t}-\omega \Delta t)\;(\mathrm{Backward}) \end{array}$$

According to Equation (9), the stair error of the azimuth caused by the asynchrony is shown in Equation (10). The stair error is proportional to the angular velocity and the asynchrony:

(10) Δϕ=2ωΔt

### 3.4. Verification Experiment

**Figure 8:**
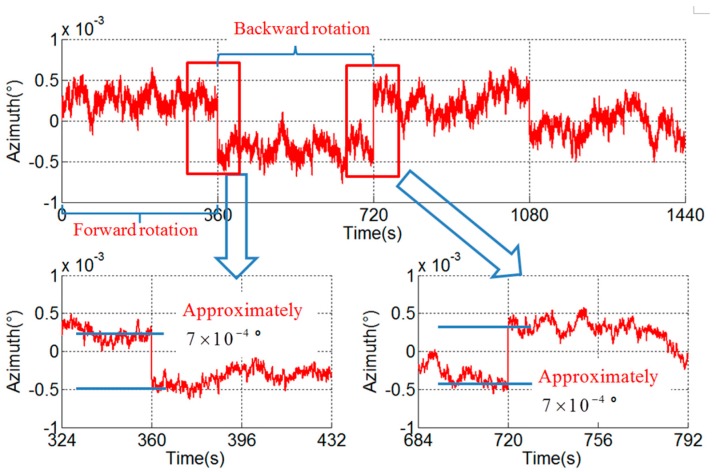
Stair error of azimuth (ω = 1°/s).

**Figure 9:**
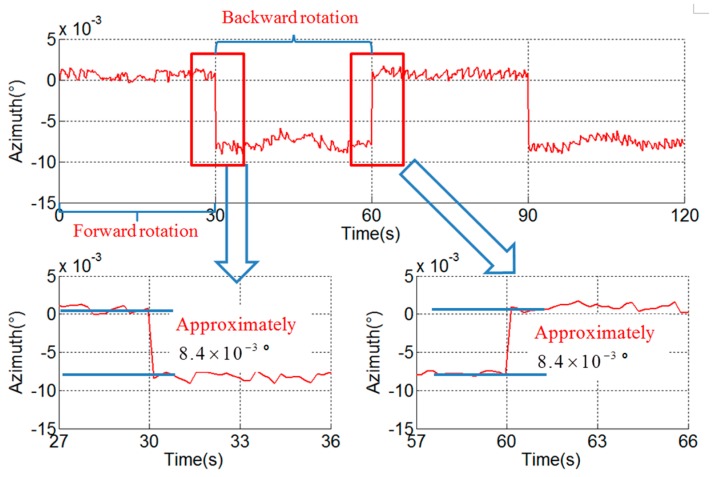
Stair error of azimuth (ω = 12°/s).

In order to distinguish the hysteresis error and the asynchrony between the angular encoder and the gyroscope, a verification experiment with different angular velocities is conducted. The angular velocities of the experiment in Figure 6, Figure 8 and Figure 9 are 6°/s, 1°/s and 12°/s respectively, and the stair errors are $4.2\times 10^{-3}$°, $7.0\times 10^{-4}$° and $8.4\times 10^{-3}$° respectively.

From the experimental results, it can be seen that there is a linear relationship between the range of the stair error and the angular velocity. This phenomenon corresponds to the characteristics of the stair error caused by asynchrony and it can be concluded that the asynchrony is the cause and the hysteresis error is negligible.

According to the analysis above and Equation (10), the asynchrony between the gyroscope and angular encode can be solved. The asynchrony is $3.5\times 10^{-4}$ s in the experimental RINS.

**Figure 10:**
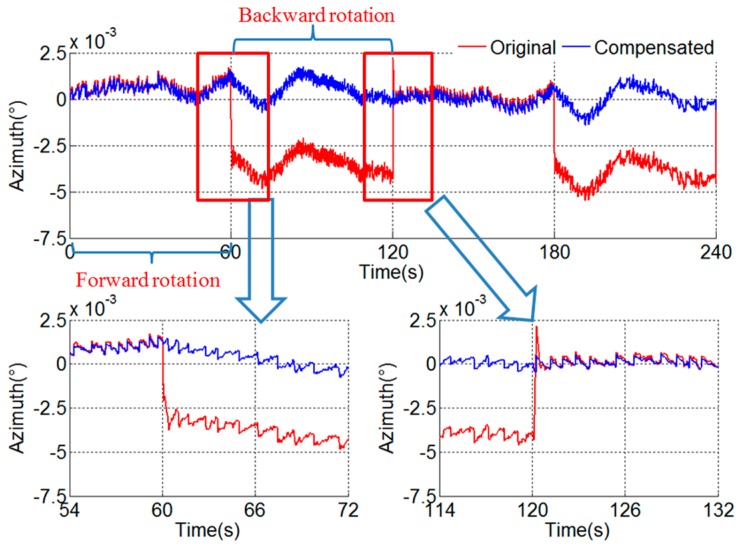
Result of compensation of asynchrony between gyroscope and angular encode.

The angular encoder outputs are delayed Δt to compensate the asynchrony. The compensated result is shown in Figure 10. The stair error of the azimuth during the turn-round of the IMU disappears after compensation.

The stair error of the azimuth between forward rotation and backward rotation is 2ωΔt and it is proportional to the angular velocity. For some rotation schemes with faster angular velocity, the impact of asynchrony will be greater. Furthermore, the changes of the angular velocity of the vehicles will lead to the attitude error because of the asynchrony based on the same principle. To some large maneuver vehicles, such as missiles, this attitude error is considerable and non-negligible. The calibration of the asynchrony between the gyroscope and angular encode is crucial for the RINS used on those vehicles.

## 4. Analysis of Asynchrony between Gyroscope and Accelerometer

**Figure 11:**
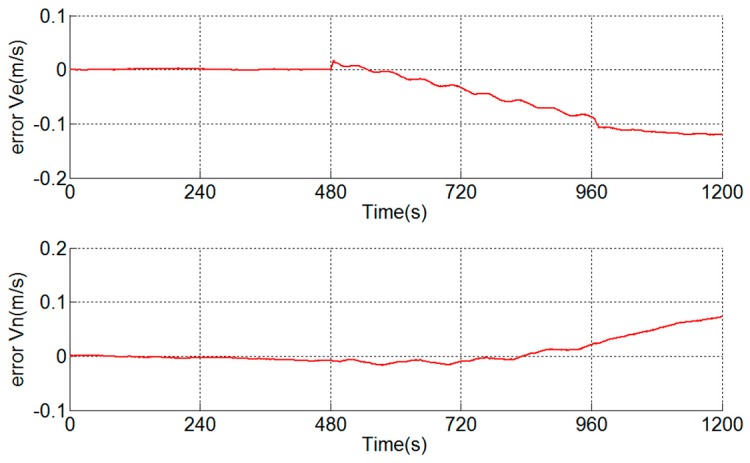
Velocity errors.

A navigation experiment is conducted under the same condition as Figure 4 and the velocity errors are shown in Figure 11. Error Ve and error Vn represent east velocity error and north velocity error, respectively. As shown in [15,16], the scale factor error of the *y*-axis of the gyroscope, misalignment angles of gyroscopes and accelerometers and inner level-arm errors can lead to velocity errors.

**Figure 12:**
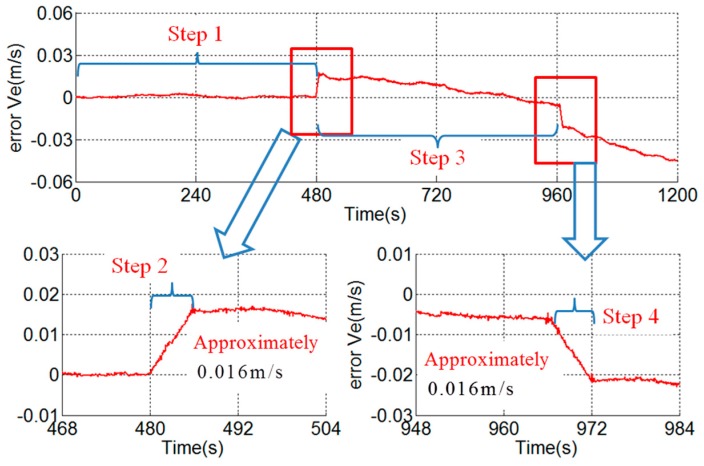
Stair error of east velocity.

After the compensation of the known error sources analyzed in the existing articles, the east velocity error is shown in Figure 12. A stair error appears on the east velocity curve during the rotation along the outer motor axis. The stair is about 0.016 m/s and is unacceptable in such a high-precision dual-axis RINS. For example, the accuracies of the gyroscopes and accelerometers of the RINS proposed in [15,28] are similar to Table 1. The root mean square of position error of the RINS is about 1.47 n mile in 50 h. In fact, the RINS in this paper will reach the similar accuracy after the compensation of the known error sources. Then the 0.016 m/s stair error is non-negligible and worth to analyze profoundly. However, there is no existing article explaining the reason for the stair error.

### 4.1. Quantitative Analysis

The stair error exists during the second and fourth steps of the rotation scheme. At those two steps, as shown in Figure 3, the IMU rotates along axis $y_{u}$ for 180°and axis $y_{u}$ points to the north in the experiment.

As shown in Figure 12, the slope of the stair error is approximately a constant and the slope can be obtained by Equation (11), where Δv equals to 0.016 m/s and t represents the time of the second step of the rotation scheme, i.e., 6 s. The slope is $2.67\times 10^{-3}{m/s}^{2}$, namely 272 μg.

(11) $$\Delta a=\frac{\Delta v}{t}$$

The east velocity stair error is equivalent to 272 μg east acceleration error. As show in Table 1, the bias of accelerometer is about 20 μg, it is one order of magnitude smaller than the equivalent east acceleration error. The northward attitude error has the same impact with the east acceleration error. The equivalent northward attitude error can be obtained by Equation (12), where Δγ represents the attitude error, $g_{0}$ represents the gravity constant. The equivalent northward attitude error is about $1.56\times 10^{-2}$°.

(12) $$\Delta \gamma =\frac{\Delta a}{g_{0}}$$

Based on the inertial navigation algorithm [27], $1.56\times 10^{-2}$° initial northward attitude error will lead to a Schuler cycle velocity error with amplitude 2.24 m/s. The latter is applicable when there are no other errors in the navigation process. That is to say, the $1.56\times 10^{-2}$° northward attitude error is very unusual whose cause is still unknown.

### 4.2. Scale Factor Error

The y-axis of the gyroscope is aligned with north during the second step of the rotation scheme in the experiment. The scale factor error of the *y*-axis of the gyroscope will lead to northward attitude error. The relationship between attitude error and scale factor error is given by Equation (13), where $\Delta k_{gy}$ represents scale factor error of the *y*-axis of the gyroscope, ω indicates the angular velocity, i.e., 30°/s, and t indicates the rotation time:

(13) $$\Delta \gamma =\Delta k_{gy}\omega t$$

As shown in Equation (13), the attitude error is proportional to the rotation time and it is not a constant during the rotation. Otherwise, the northward attitude error caused by scale factor error will greatly affect the velocity error during the third step of t:e rotation scheme too, and eventually leads to 2.24 m/s Schuler cycle velocity error [27]. The velocity error of the third step of the rotation scheme is basically normal as shown in Figure 12, so it is not the scale factor error of the *y*-axis of the gyroscope that causes the velocity stair error.

### 4.3. Asynchrony between Gyroscope and Accelerometer

The equivalent $1.56\times 10^{-2}$° northward attitude error exists only when the IMU rotates around the axis pointing northward. A hypothesis is that the asynchrony between the gyroscope and the accelerometer is a cause for the attitude error. This asynchrony error will not lead to equivalent velocity error as the IMU rotates around the axis pointing skyward. But when it comes to rotation along horizontal axis, it will lead to great equivalent velocity error. The velocity error caused by asynchrony error will be analyzed in theory below.

**Figure 13:**
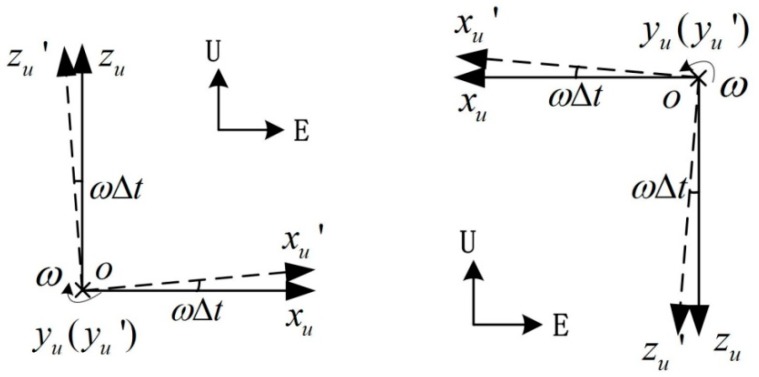
IMU frame at time *t* and *t* − Δ*t*.

It is supposed that the gyroscope data is real-time, while the accelerometer data is Δt delayed. This means that the gyroscope data indicate the angular velocity at time t, while the accelerometer data indicate the acceleration at time t−Δt. As shown in Figure 13a, $o-x_{u}y_{u}z_{u}$ frame is the IMU frame at time t, while $o-x_{u}^{\prime }y_{u}^{\prime }z_{u}^{\prime }$ frame is the IMU frame at time t−Δt.

The RINS is placed East-North-Up, the outer motor axis is aligned with the north direction. As shown in Figure 13a, during the second step of the rotation scheme, axis $y_{u}$ is aligned with the outer motor axis. The acceleration along axis $x_{u}$ and $z_{u}$ is given by Equation (14); while the outputs of accelerometers are the acceleration along $x_{u}^{\prime }$ and $z_{u}^{\prime }$, which is given by Equation (15):

(14) $$\left\{ \begin{array}{l} a_{x}=g\sin\gamma_{t}\\ a_{z}=-g\cos\gamma_{t} \end{array}\right.$$

(15) $$\left\{ \begin{array}{l} a_{x}^{\prime }=g\sin(\gamma_{t}-\omega \Delta t)\\ a_{z}^{\prime }=-g\cos(\gamma_{t}-\omega \Delta t) \end{array}\right.$$

where $\gamma_{t}$ indicates the roll angle of the IMU, and g indicates gravity.

By combining Equations (14) and (15), the acceleration errors along axis $x_{u}$ and $z_{u}$ during the second step of the rotation scheme is given by Equation (16):

(16) $$\left\{ \begin{array}{l} \Delta a_{x}=g\sin(\gamma_{t}-\omega \Delta t)-g\sin\gamma_{t}=g\sin\gamma_{t}\cos\omega \Delta t-g\cos\gamma_{t}\sin\omega \Delta t-g\sin\gamma_{t}\\ \Delta a_{z}=-g\cos(\gamma_{t}-\omega \Delta t)+g\cos\gamma_{t}=-g\cos\gamma_{t}\cos\omega \Delta t-g\sin\gamma_{t}\sin\omega \Delta t+g\cos\gamma_{t} \end{array}\right.$$

As ωΔt is a small angle, Equation (16) can be simplified to Equation (17):

(17) $$\left\{ \begin{array}{l} \Delta a_{x}=-g\omega \Delta t\cos\gamma_{t}\\ \Delta a_{z}=-g\omega \Delta t\sin\gamma_{t} \end{array}\right.$$

$\Delta a_{x}$ and $\Delta a_{z}$ are projected into navigation frame and the acceleration errors along the navigation frame are obtained, as shown in Equation (18). The asynchrony between the gyroscope and accelerometer will lead to a constant east acceleration error during the rotation along the outer motor axis, causing the stair error of the east velocity:

(18) $$\left\{ \begin{array}{l} \Delta a_{E}=\Delta a_{x}\cos\gamma_{t}+\Delta a_{z}\sin\gamma_{t}=(-g\omega \Delta t\cos\gamma_{t})\cos\gamma_{t}+(-g\omega \Delta t\sin\gamma_{t})\sin\gamma_{t}=-g\omega \Delta t\\ \Delta a_{U}=-\Delta a_{x}\sin\gamma_{t}+\Delta a_{z}\cos\gamma_{t}=-(-g\omega \Delta t\cos\gamma_{t})\sin\gamma_{t}+(-g\omega \Delta t\sin\gamma_{t})\cos\gamma_{t}=0 \end{array}\right.$$

The stair error of east velocity during the second step of the rotation scheme is given by Equation (19):

(19) Δv=−gωΔtT

where Δv indicates the stair error during the second step of the rotation scheme, ω indicates the angular velocity, i.e., 30°/s, and T indicates the period of the second step of the rotation scheme, i.e., 6 s. The ωT in Equation (19) is the angle rotated, i.e., 180° in the experiment. The stair error of east velocity is proportional to the angle rotated.

### 4.4. Verification Experiment

As shown in Figure 12, the velocity error in the fourth step is opposite to that in the second step. The fourth step of the rotation scheme is shown in Figure 13b. In the similar way to the analysis above, the east velocity error stair caused by the asynchrony is given by Equation (20). The error stairs of the second step and the fourth step are opposite, which is consistent with the velocity error in Figure 12:

(20) Δv=gωΔtT

**Figure 14:**
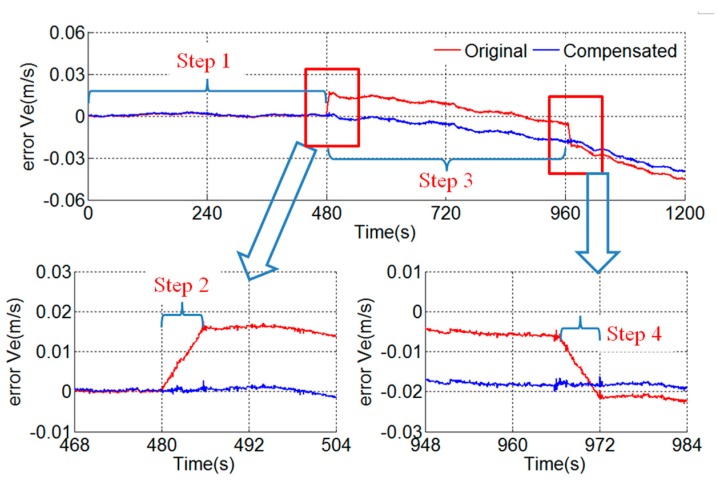
Result of compensation of asynchrony between gyroscope and accelerometer.

Based on Equation (19), the asynchrony between the gyroscope and accelerometer can be obtained by Equation (21). The asynchrony is $5.2\times 10^{-4}$ s in the experimental RINS. Figure 14 shows the results of the compensation of the asynchrony between the gyroscope and accelerometer. The velocity stair error during the rotation along the outer motor axis disappears after the compensation:

(21) $$\Delta t=\frac{\Delta v}{g\omega T}$$

According to Equation (19), the stair error of east velocity is proportional to the angle rotated. If the rotation scheme contains a constant rotation along the outer motor axis, the stair error caused by the asynchrony will be enlarged proportionally. Furthermore, the angular maneuver of the vehicle has the same effect with the IMU rotation. With respect to some fast rotating vehicles, such as spinning missiles, the impact of the asynchrony will be enlarged in proportion to the angle rotated.

## 5. Conclusions

The asynchrony between the gyroscope and angular encoder and the asynchrony between the gyroscope and accelerometer are analyzed in this paper. Based on the azimuth stair of the RINS during the turn-round along the inner motor axis, the asynchrony between the gyroscope and angular encoder is calibrated. The stair error of the azimuth during the turn-round of the IMU disappears after compensation. The asynchrony between the gyroscope and accelerometer leads to the stair error on the velocity curve, and is unacceptable in a high-precision RINS. Based on the velocity stair of the RINS during the rotation along the outer motor axis, the asynchrony is calibrated. The velocity stair error disappears after compensation. In addition, the proposed self-calibration algorithm is a general method, and is not only suitable for the dual-axis RINS analyzed in this paper, but also for other types of RINS or even SINS. Furthermore, for the INS used in large maneuver vehicle or fast rotating vehicle, the effect of asynchrony will be greatly magnified, and thus the method proposed in this paper will be even more significant.

## Figures and Tables

**Table 1:** Specifications of main components of RINS.

Gyroscope bias	0.005°/h
Accelerometer bias	20 μg
Encoder resolution	5.278 × 10^6^ °/pulse
Calculation frequency	200 Hz
